# Supplementary material for: Molecular detection of Coxiella burnetii in raw meat samples collected from different abattoirs in districts Kasur and Lahore of Punjab, Pakistan
Source: PLoS One. 2023 Aug 11;18(8):e0289944. doi: 10.1371/journal.pone.0289944 (PMC10420375; doi:10.1371/journal.pone.0289944)
Supplement: S1 Table — (DOCX) [file pone.0289944.s001.docx]

**Supplementary Table S1** Univariable analysis of *Coxiella burnetii* in raw meat samples collected from slaughter houses in districts Kasur and Lahore in Punjab province, Pakistan, 2021-2022

| **Variable** | **Prevalence (%)** | **P value** |
| --- | --- | --- |
| 1. **District** |  | 1.000 |
| Lahore | 20 |  |
| Kasur | 20 |  |
| 1. **Species** |  | 0.0008 |
| Cattle | 12 |  |
| Buffalo | 8 |  |
| Goat | 22 |  |
| Sheep | 38 |  |
| 1. **Meat Type** |  | 0.0004 |
| Mutton | 30 |  |
| Beef | 10 |  |
| 1. **Body condition** |  | 0.35 |
| Good | 0 |  |
| Weak | 0 |  |
| Average | 20 |  |
